# Explicit bounds of unknown function of some new weakly singular retarded integral inequalities for discontinuous functions and their applications

**DOI:** 10.1186/s13660-017-1563-0

**Published:** 2017-11-17

**Authors:** Zizun Li, Wu-Sheng Wang

**Affiliations:** 1 School of Mathematics, Sichuan University, Chengdu, Sichuan 610064 P.R. China; 2 School of Mathematics and Statistics, Baise University, Baise, Guangxi 533000 P.R. China; 3 School of Mathematics and Statistics, Hechi University, Yizhou, Guangxi 546300 P.R. China

**Keywords:** 26D10, 26D15, 26D20, 45A99, integral inequality for discontinuous function, retarded, weakly singular, explicit bounds, singular integral equation

## Abstract

The purpose of the present paper is to establish some new retarded weakly singular integral inequalities of Gronwall-Bellman type for discontinuous functions, which generalize some known weakly singular and impulsive integral inequalities. The inequalities given here can be used in the analysis of the qualitative properties of certain classes of singular differential equations and singular impulsive equations.

## Introduction

Being an important tool in the study of qualitative properties of solutions of differential equations and integral equations, various generalizations of Gronwall-Bellman integral inequality and their applications have attracted great interest of many mathematicians (such as [1–11] and the references therein). Gronwall [11] and Bellman [5] established the integral inequality

$$ u(t)\leq c+ \int^{t}_{a} f(s)u(s)\,ds, \quad t\in[a, b], $$

for some constant $c\geq0$, obtained the estimation of an unknown function,

$$\begin{aligned} u(t)\leq c\exp \biggl( \int^{t}_{a} f(s)\,ds \biggr), \quad t\in[a, b]. \end{aligned}$$

Abdeldaim [12] discussed the following nonlinear integral inequality:

$$\begin{aligned}& u(t) \le u_{0}+ \int_{0}^{\alpha(t)}f(s) \biggl[u^{2-p}(s)+ \int^{s}_{0}g(\tau )u^{q}(\tau)\,d\tau \biggr]^{p}\,ds,\quad p\in[0,1), \\& u(t) \le n(t)+ \int_{0}^{\alpha(t)}f(s) \biggl[u(s)+ \int^{s}_{0}g(\tau)u(\tau )\,d\tau \biggr]^{p}\,ds, \quad p\in[0,1). \end{aligned}$$

Usually, this type integral inequalities have regular or continuous integral kernels, but some problems of theory and practicality require us to solve integral inequalities with singular kernels. For example, to prove a global existence and an exponential decay result for a parabolic Cauchy problem. Henry [13] investigated the following linear singular integral inequality:

$$ u(t) \le a+b \int_{0}^{t}(t-s)^{\beta-1}u(s)\,ds. $$

Sano and Kunimatsu[14] generalized Henry’s type inequality to

$$ 0\le u(t) \le c_{1}+c_{2}t^{\alpha-1}+c_{3} \int_{0}^{t}u(s)\,ds+c_{4} \int _{0}^{t}(t-s)^{\beta-1}u(s)\,ds, $$

and gave a sufficient condition for stabilization of semilinear parabolic distributed systems. Ye *et al.* [15] discussed the linear singular integral inequality

$$ u(t) \le a(t)+b(t) \int_{0}^{t}(t-s)^{\beta-1}u(s)\,ds, $$

and they used it to study the dependence of the solution and the initial condition to a certain fractional differential equation with Riemann-Liouville fractional derivatives. All inequalities of this type are proved by an iteration argument and the estimation formulas are expressed by a complicated power series which is sometimes not very convenient for applications. To avoid the weakness, Medveď [16] presented a new method to solve integral inequalities of Henry-Gronwall type, then he got the explicit bounds with a quite simple formula, similar to the classic Gronwall-Bellman inequalities. Furthermore, he also obtained global solutions of the semilinear evolutions in [17]. In 2008, Ma and Pečarić [18] used the modification of Medveď’s method to study a new weakly singular integral inequality,

$$ u^{p}(t) \le a(t) +b(t) \int_{0}^{t}\bigl(t^{\beta}-s^{\beta} \bigr)^{\gamma-1} s^{\xi -1}f(s)u^{q}(s)\,ds,\quad t\in[0, + \infty). $$

Besides the results mentioned above, various investigators have discovered many useful and new weakly singular integral inequalities, mainly inspired by their applications in various branches of fractional differential equations (see [14, 16–27] and the references therein).

In analyzing the impulsive phenomenon of a physical system governed by certain differential and integral equations, by estimating the unknown function in the integral inequality of the discontinuous functions, Some properties of the solution of some impulsive differential equations can be studied. These inequalities and their various linear and nonlinear generalizations are crucial in the discussion of the existence, uniqueness, boundedness, stability, and other qualitative properties of solutions of differential and integral equations (see [10, 25, 28–34] and the references therein). Tatar [25] discussed the following class of integral inequalities:

$$\begin{aligned}& u(t)\le a(t)+b(t) \int^{t}_{0}k_{1}(t,s)u^{m}(s) \,ds + c(t) \int ^{t}_{0}k_{2}(t,s)u^{n}(s- \tau)\,ds \\& \hphantom{u(t)\le{}}{}+d(t)\sum_{0< t_{k}< t}\eta_{k}u(t_{k}), \quad t\ge0, \\& u(t) \le \varphi(t), \quad t\in[-\tau,0], \tau>0, \end{aligned}$$

where $k_{i}(t,s)=(t-s)^{\beta_{i}-1}s^{\gamma_{i}}F_{i}(s)$, $i=1,2$. Iovane [28] studied the following discontinuous function integral inequality:

$$\begin{aligned} u(t) \le a(t) + \int_{t_{0}}^{t} f(s)u\bigl(\tau(s)\bigr)\,ds+\sum _{t_{0}< t_{i}< t}\beta_{i}u^{m}(t_{i}-0), \quad \forall t\ge t_{0}, \end{aligned}$$

where $a(t)>0$, $f(t)\ge0$, $g(t)\ge0$, $\beta_{i}\ge0$, $m>0$. Gllo *et al.* [10] studied the impulsive integral inequality

$$ u(t) \le a(t) + g(t) \int_{t_{0}}^{t} q(s)u^{n}\bigl(\tau(s)\bigr) \,ds+p(t)\sum_{t_{0}< t_{i}< t}\beta_{i}u^{m}(t_{i}-0), \quad \forall t\ge t_{0}, $$

where $a(t)$ is a nondecreasing function as $t\ge t_{0}$, $g(t)\ge 1$, $p(t)\ge1$, $q(s)\in C(\mathbf{R_{+}},\mathbf{R_{+}})$, $\tau:\mathbf{R}\rightarrow\mathbf{R}$, $\tau(s)\le s$, $\lim_{|s|\rightarrow\infty}\tau(s)=\infty$, $\beta_{i}\ge0 $, $m>0$. Yan [32] discussed the impulsive integral inequality with delay

$$\begin{aligned} u(t) \le& a(t) + \int_{t_{0}}^{t} f(t,s)u\bigl(\tau(s)\bigr)\,ds+ \int_{t_{0}}^{t} f(t,s) \biggl( \int_{t_{0}}^{s} g(s,\theta )u\bigl(\tau(\theta)\bigr)d \theta \biggr)\,ds \\ &{}+q(t)\sum_{t_{0}< t_{i}< t}\beta_{i}u^{m}(t_{i}-0), \quad \forall t \ge t_{0}, \end{aligned}$$

where $a(t)\in C(\mathbf{R_{+}},\mathbf{R_{+}})$, $f,g\in C(\mathbf {R^{2}_{+}},\mathbf{R_{+}})$, $\tau:\mathbf{R}\rightarrow\mathbf{R}$, $\tau(s)\le s$, $\lim_{|s|\rightarrow\infty}\tau(s)=\infty$, $\beta_{i}\ge0 $, $m>0$. Mi *et al.* [30] studied the integral inequality of complex functions with unknown function

$$\begin{aligned} u(t) \le& a(t) + \int_{t_{0}}^{t} f(t,s) \int_{t_{0}}^{s} g(s,\tau)w\bigl(u(\tau )\bigr)\,d\tau \,ds \\ &{}+q(t)\sum_{t_{0}< t_{i}< t}\beta_{i}u^{m}(t_{i}-0), \quad \forall t \ge t_{0}, \end{aligned}$$

where $w(u)$ is monotone decreasing continuous function defined on $[0,\infty)$, and $w(u)>0$ when $u>0$. Liu *et al.* [29] investigated the impulsive integral inequality with delay

$$ u^{p}(t) \le a(t) + b(t) \int_{t_{0}}^{t} \bigl[f(s)u^{q}(s)+h(s)u^{r} \bigl(\sigma(s)\bigr)\bigr]\,ds+\sum_{t_{0}< t_{i}< t}\beta _{i}u^{m}(t_{i}-0), \quad \forall t\ge t_{0}, $$

where $a(t),b(t)\ge1$ are both nondecreasing functions at $t\ge t_{0}$, $f(s),h(s)\in C(\mathbf{R_{+}},\mathbf{R_{+}})$, $\sigma(s)\le s$, $\lim_{|s|\rightarrow\infty}=\infty$, $\beta_{i}\ge0$, $m>0$, $p\ge q\ge0$, $p\ge r\ge0$. Zheng *et al.* [34] studied the following integral inequality for discontinuous function:

$$\begin{aligned} u^{p}(t) \le& a_{0}(t)+\frac{p}{p-1}\sum _{i=1}^{N} \int^{t}_{t_{0}} g_{i}(s)u^{q} \bigl(\phi_{i}(s)\bigr)\,ds +\sum^{L}_{j=1} \int^{t}_{t_{0}}b_{j}(s) \int^{s}_{t_{0}}c_{j}(\theta)u^{q} \bigl(w_{j}(\theta )\bigr)d\theta \,ds \\ &{}+\sum_{t_{0}< t_{i}< t}\beta_{i} u^{q}(t_{i}-0), \end{aligned}$$

where $u(t)$, $a(t)$ and $g_{i}(t)$, $b_{j}(t)$, $c_{j}(t)$ ($1\le i\le N$, $1\le j\le L$) are positive and continuous functions on $[t_{0},\infty)$, and $c_{j}(t)$ are nondecreasing functions on $[t_{0},\infty)$, and $\phi _{i}(t)$, $w_{j}(t)$ are continuous functions on $[t_{0},\infty)$ and $t_{0}\le\phi_{i}(t)\le t$, $t_{0}\le w_{j}(t)\le t$.

However, in certain situations, such as some classes of delay impulsive differential equations and delay impulsive integral equations, it is desirable to find some new delay impulsive inequalities, in order to achieve a diversity of desired goals. In this paper, we discuss a class of retarded integral inequalities with weak singularity for discontinuous functions,

1 $$\begin{aligned}& u(t) \le a(t)+ \int_{0}^{\alpha(t)}\bigl(\alpha^{\beta}(t)-s^{\beta} \bigr)^{\gamma -1} s^{\xi-1}f_{1}(s)u(s)\,ds \\& \hphantom{u(t) \le{}}{}+ \int_{0}^{\alpha(t)}\bigl(\alpha^{\beta}(t)-s^{\beta} \bigr)^{\gamma-1} s^{\xi -1}f_{2}(s) \int^{s}_{0} f_{3}(\tau)u(\tau)\,d\tau \,ds, \quad t\in\mathbf{R}_{+}, \end{aligned}$$

2 $$\begin{aligned}& u(t) \le a(t)+ \int_{t_{0}}^{\alpha(t)}\bigl(t^{\beta}-s^{\beta} \bigr)^{\gamma -1}f(s)u(s) \biggl[u^{2}(s)+ \int^{s}_{t_{0}}g(\tau)u(\tau)\,d\tau \biggr]^{p}\,ds \\& \hphantom{u(t) \le{}}{}+\sum_{t_{0}< t_{i}< t}\beta_{i}u(t_{i}-0), \end{aligned}$$

3 $$\begin{aligned}& u^{p}(t) \le a(t)+b(t) \int_{t_{0}}^{\alpha(t)}\bigl(\alpha^{\beta}(t)-s^{\beta } \bigr)^{\gamma-1} s^{\xi-1}f(s) \biggl[u^{m}(s)+ \int^{s}_{t_{0}}g(\tau)u^{n}(\tau )\,d\tau \biggr]^{q}\,ds \\& \hphantom{u^{p}(t) \le{}}{}+\sum_{t_{0}< t_{i}< t}\beta_{i}u^{p}(t_{i}-0), \end{aligned}$$

which generalize the inequality (2) in [12] to the weakly singular integral inequality, and (4) in [18] to the retarded inequality. We use the modification of Medveď’s method to obtain the explicit estimations of the unknown function in the inequality (1), and we use the analysis technique to get the explicit estimations of the unknown function in the inequalities (2) and (3). Finally, we give two examples to illustrate applications of our results.

## Main results

Throughout this paper, **R** denotes the set of real numbers and $\mathbf{R_{+}}=[0,\infty)$ is the given subset of **R**, and $C(M,S)$ denotes the class of all continuous functions defined on set *M* with range in the set *S*.

The following lemmas are very useful in the procedures of our proof in our main results.

### Lemma 1

*Suppose that* $f(x)$ *and* $g(x)$ *are nonnegative and continuous functions on* $[c,d]$. *Let* $p>1$, $\frac{1}{q}+\frac{1}{p}=1$. *Then*

4 $$ \int_{c}^{d}f(s)g(s)\,ds\le \biggl( \int_{c}^{d}f^{p}(s)\,ds \biggr)^{1/p} \biggl( \int _{c}^{d}g^{q}(s)\,ds \biggr)^{1/q}. $$

*Let* $\alpha(t)$ *be a continuous*, *differentiable and increasing function on* $[t_{0},+\infty)$ *with* $\alpha(t)\le t, \alpha(t_{0})= t_{0}$, *then*

5 $$ \int_{\alpha(t_{0})}^{\alpha(t)}f(s)g(s)\,ds \le \biggl( \int_{\alpha (t_{0})}^{\alpha(t)}f^{p}(s)\,ds \biggr)^{1/p} \biggl( \int_{\alpha(t_{0})}^{\alpha (t)} g^{q}(s) \,ds \biggr)^{1/q}. $$

### Proof

We prove the inequality (5). Using the inequality (4), we obtain

$$\begin{aligned} \int_{\alpha(t_{0})}^{\alpha(t)}f(s)g(s)\,ds =& \int_{t_{0}}^{t}f\bigl(\alpha (s)\bigr)g\bigl( \alpha(s)\bigr)\alpha'(s)\,ds= \int_{t_{0}}^{t}f\bigl(\alpha(s)\bigr) \bigl(\alpha '(s)\bigr)^{1/p}g\bigl(\alpha(s)\bigr) \bigl( \alpha'(s)\bigr)^{1/q}\,ds \\ \le& \biggl( \int_{t_{0}}^{t}f^{p}\bigl(\alpha(s)\bigr) \alpha'(s)\,ds \biggr)^{1/p} \biggl( \int _{t_{0}}^{t}g^{q}\bigl(\alpha(s)\bigr) \alpha'(s)\,ds \biggr)^{1/q} \\ =& \biggl( \int_{\alpha(t_{0})}^{\alpha(t)}f^{p}(s)\,ds \biggr)^{1/p} \biggl( \int _{\alpha(t_{0})}^{\alpha(t)} g^{q}(s) \,ds \biggr)^{1/q}. \end{aligned}$$

 □

### Lemma 2

([35])

*Let* $a_{1}, a_{2},\ldots,a_{n}$ *be nonnegative real numbers*, $m>1$ *is a real number*, *and* *n* *is a natural number*. *Then*

6 $$ (a_{1}+a_{2}+\cdots+a_{n})^{m} \le n^{m-1}\bigl(a_{1}^{m}+a_{2}^{m}+ \cdots+a_{n}^{m}\bigr). $$

### Lemma 3

([18, 21])

*Let* *β*, *γ*, *ξ* *and* *p* *be positive constants*. *Then*

$$ \int_{0}^{t}\bigl(t^{\beta}-s^{\beta} \bigr)^{p(\gamma-1)} s^{p(\xi-1)}\,ds=\frac {t^{\theta}}{\beta}B \biggl[ \frac{p(\xi-1)+1}{\beta},p(\gamma-1)+1 \biggr],\quad t\in[0,+\infty). $$

*Let* $\alpha(t)$ *be a continuous*, *differentiable and increasing function on* $[t_{0},+\infty)$ *with* $\alpha(t)\le t$, $\alpha(t_{0})= t_{0}$, *then*

$$ \int_{\alpha(t_{0})}^{\alpha(t)}\bigl(\alpha^{\beta}(t)-s^{\beta} \bigr)^{p(\gamma -1)} s^{p(\xi-1)}\,ds\le\frac{\alpha^{\theta}(t)}{\beta}B \biggl[ \frac{p(\xi -1)+1}{\beta},p(\gamma-1)+1 \biggr],\quad t\in[0,+\infty), $$

*where* $B[x,y]=\int^{1}_{0} s^{ x-1}(1-s)^{y-1}\,ds$ ($x>0$, $y>0$) *is the well*-*known beta*-*function and* $\theta=p[\beta(\gamma-1)+\xi-1]+1$. *Suppose that the positive constants* *β*, *γ*, *ξ*, $p_{1}$ *and* $p_{2}$ *satisfy conditions*:

1. *if* $\beta\in(0,1],\gamma\in(1/2,1)$ *and* $\xi\ge3/2-\gamma ,p_{1}=1/\gamma$;
2. *if* $\beta\in(0,1],\gamma\in(0,1/2]$ *and* $\xi> (1-2\gamma ^{2})/(1-\gamma^{2}), p_{2}=(1+4\gamma)/(1+3\gamma)$, *then*
  $$ B \biggl[\frac{p_{i}(\xi-1)+1}{\beta},p_{i}(\gamma-1)+1 \biggr]\in[0,+ \infty), $$
  *and* $\theta_{i}=p_{i}[\beta(\gamma-1)+\xi-1]+1\ge0$ *are valid for* $i=1,2$.

### Lemma 4

*Let* $u(t),a(t),b(t),h(t)\in C(\mathbf{R}_{+},\mathbf{R}_{+})$, $\alpha(t)$ *be a continuous*, *differentiable and increasing function on* ${\mathbf{R}_{+}}$ *with* $\alpha(t)\le t$, $\alpha(0)= 0$. *If* $u(t)$ *satisfies the following inequality*:

7 $$ u(t) \le a(t)+b(t) \int_{0}^{\alpha(t)}h(s)u(s)\,ds. $$

*Then*

8 $$ u(t) \le a(t)+\frac{b(t)}{e(\alpha(t))} \int_{0}^{\alpha(t)}h(s)a(s)e(s)\,ds, $$

*where*

9 $$ e(t) = \exp \biggl(- \int_{0}^{t}h(s)b(s)\,ds \biggr). $$

### Proof

Define a function $v(t)$ on ${\mathbf{R}_{+}}$ by

10 $$ v(t) = e\bigl(\alpha(t)\bigr) \int_{0}^{\alpha(t)}h(s)u(s)\,ds, $$

we have $v(0)=0$. Differentiating $v(t)$ with respect to *t* and using (7) and (9), we have

11 $$\begin{aligned} v'(t) =&\alpha'(t)h\bigl(\alpha(t)\bigr)u\bigl( \alpha(t)\bigr)e\bigl(\alpha(t)\bigr)-\alpha '(t)h\bigl(\alpha(t) \bigr)b\bigl(\alpha(t)\bigr)e\bigl(\alpha(t)\bigr) \int_{0}^{\alpha (t)}h(s)u(s)\,ds \\ \le&\alpha'(t)h\bigl(\alpha(t)\bigr)a\bigl(\alpha(t)\bigr)e\bigl( \alpha(t)\bigr)+\alpha'(t)h\bigl(\alpha (t)\bigr)e\bigl(\alpha(t) \bigr)b\bigl(\alpha(t)\bigr) \int_{0}^{\alpha(t)}h(s)u(s)\,ds \\ &{}-\alpha'(t)h\bigl(\alpha(t)\bigr)b\bigl(\alpha(t)\bigr)e\bigl( \alpha(t)\bigr) \int_{0}^{\alpha (t)}h(s)u(s)\,ds \\ \le&\alpha'(t)h\bigl(\alpha(t)\bigr)a\bigl(\alpha(t)\bigr)e\bigl( \alpha(t)\bigr). \end{aligned}$$

Integrating both sides of the inequality (11) from 0 to *t*, since $v(0)=0$ we get

12 $$ v(t)\le \int^{t}_{0}\alpha'(s)h\bigl(\alpha(s) \bigr)a\bigl(\alpha(s)\bigr)e\bigl(\alpha(s)\bigr)\,ds= \int ^{\alpha(t)}_{0}h(s)a(s)e(s)\,ds. $$

From (10) and (12), we obtain

13 $$ \int_{0}^{\alpha(t)}h(s)u(s)\,ds \le \frac{1}{e(\alpha(t))} \int^{\alpha(t)}_{0}h(s)a(s)e(s)\,ds. $$

Substituting the inequality (13) into (7) we get the required estimation (8). The proof is completed. □

### Lemma 5

*Let* $a\ge0$, $p\ge q\ge0$ *and* $p\neq0$, *then*

14 $$ a^{\frac{q}{p}}\le\frac{q}{p}a+\frac{p-q}{p}. $$

### Proof

If $q=0$, the inequality above is obviously valid. On the other hand, if $q>0$, let $\delta=q/p$, then $\delta\le1$, by [36], [18] (Lemma 2.1), we obtain

$$ a^{\frac{q}{p}}\le\frac{q}{p}K^{(q-p)/p}a+ \frac{p-q}{p}K^{q/p}, $$

for any $K>0$. Let $K=1$, we get (14). □

### Theorem 1

*Let* $a(t), f_{1} (t),f_{2}(t), f_{3}(t)\in C(\mathbf{R}_{+},\mathbf{R}_{+})$, *and* $a(t)$ *is a nondecreasing function*, *and let* $\alpha(t)$ *be a continuous*, *differentiable and increasing function on* ${\mathbf{R}_{+}}$ *with* $\alpha(t)\le t$, $\alpha(0)= 0$. *Let* $\beta,\gamma,\xi$ *be positive constants*. *Suppose that* $u(t)$ *satisfies the inequality* (1).

(1) *If* $\beta\in(0,1]$, $\gamma\in(1/2,1)$ *and* $\xi\ge3/2-\gamma$, *we have*

15 $$ u(t)\le \biggl(\tilde{a}_{1}(t)+\frac{\tilde{b}_{1}(t)}{\tilde{e}_{1}(\alpha (t))} \int_{0}^{\alpha(t)}\tilde{h}_{1}(s) \tilde{a}_{1}(s)\tilde {e}_{1}(s)\,ds \biggr)^{1-\gamma}, \quad t\in{\mathbf{R}_{+}}, $$

*where*

$$\begin{aligned}& \tilde{a}_{1}(t) = 3^{\frac{\gamma}{1-\gamma}}a^{\frac{1}{1-\gamma}}(t), \\& \tilde{b}_{1}(t) = \bigl(3M_{1} \alpha^{\theta_{1}}(t) \bigr)^{\frac{\gamma }{1-\gamma}}, \\& \tilde{h}_{1}(t) = f_{1}^{\frac{1}{1-\gamma}}(t)+ \biggl(f_{2}(t) \int^{t}_{0}f_{3}(\tau )\,d\tau \biggr)^{\frac{1}{1-\gamma}}, \\& \tilde{e}_{1}(t) = \exp \biggl(- \int_{0}^{t}\tilde{h}_{1}(s)\tilde {b}_{1}(s)\,ds \biggr), \\& M_{1} = \frac{1}{\beta}B \biggl[\frac{\gamma+\xi-1}{\beta\gamma}, \frac {2\gamma-1}{\gamma} \biggr], \\& \theta_{1} = \frac{1}{\gamma}\bigl[\beta(\gamma-1)+\xi-1 \bigr]+1. \end{aligned}$$

(2) *If* $\beta\in(0,1],\gamma\in(0,1/2]$ *and* $\xi> (1-2\gamma ^{2})/(1-\gamma^{2})$, *we have*

16 $$\begin{aligned} w(t)\le \biggl(\tilde{a}_{2}(t)+\frac{\tilde{b}_{2}(t)}{\tilde{e}_{2}(\alpha (t))} \int_{0}^{\alpha(t)}\tilde{h}_{2}(s) \tilde{a}_{2}(s)\tilde {e}_{2}(s)\,ds \biggr)^{\frac{\gamma}{1+4\gamma}}, \quad t\in{\mathbf{R}_{+}}, \end{aligned}$$

*where*

$$\begin{aligned}& \tilde{a}_{2}(t) = 3^{\frac{1+3\gamma}{\gamma}}a^{\frac{1+4\gamma}{\gamma }}(t), \\& \tilde{b}_{2}(t) = \bigl(3M_{2} \alpha^{\theta_{2}}(t) \bigr)^{\frac{1+3\gamma }{\gamma}}, \\& \tilde{h}_{2}(t) = f_{1}^{\frac{1+4\gamma}{\gamma}}(s)+ \biggl(f_{2}(s) \int^{s}_{0} f_{3}(\tau)\,d\tau \biggr)^{\frac{1+4\gamma}{\gamma}}, \\& \tilde{e}_{2}(t) = \exp \biggl(- \int_{0}^{t}\tilde{h}_{2}(s)\tilde {b}_{2}(s)\,ds \biggr), \\& M_{2} = \frac{1}{\beta}B \biggl[\frac{\xi(1+4\gamma)-\gamma}{\beta(1+3\gamma )}, \frac{4\gamma^{2}}{1+3\gamma} \biggr], \\& \theta_{2} = \frac{1+4\gamma}{1+3\gamma}\bigl[\beta(\gamma-1)+\xi-1 \bigr]+1. \end{aligned}$$

### Proof

If $\beta\in(0,1]$, $\gamma\in(1/2,1)$ and $\xi\ge3/2-\gamma$, let

$$p_{1}=\frac{1}{\gamma},\qquad q_{1}=\frac{1}{(1-\gamma)}, $$

if $\beta\in(0,1]$, $\gamma\in(0,1/2]$ and $\xi> (1-2\gamma^{2})/(1-\gamma^{2})$, let

$$p_{2}=\frac{(1+4\gamma)}{(1+3\gamma)},\qquad q_{2}=\frac{(1+4\gamma)}{\gamma}, $$

then

$$\frac{1}{p_{i}}+\frac{1}{q_{i}}=1, \quad i=1,2. $$

Using Hölder’s inequality in Lemma 1 applied to (1), we have

$$\begin{aligned} u(t) \le& a(t)+ \biggl[ \int_{0}^{\alpha(t)}\bigl(\alpha^{\beta}(t)-s^{\beta } \bigr)^{p_{i}(\gamma-1)} s^{p_{i}(\xi-1)}\,ds \biggr]^{1/p_{i}} \biggl[ \int_{0}^{\alpha (t)}f_{1}^{q_{i}}(s)u^{q_{i}}(s) \,ds \biggr]^{1/q_{i}} \\ &{}+ \biggl[ \int_{0}^{\alpha(t)}\bigl(\alpha^{\beta}(t)-s^{\beta} \bigr)^{p_{i}(\gamma -1)} s^{p_{i}(\xi-1)}\,ds \biggr]^{1/p_{i}} \\ &{}\times \biggl[ \int_{0}^{\alpha(t)} \biggl(f_{2}(s) \int^{s}_{0} f_{3}(\tau)u(\tau)\,d\tau \biggr)^{q_{i}} \,ds \biggr]^{1/q_{i}}. \end{aligned}$$

Set

17 $$\begin{aligned} z(t) =&a(t)+ \biggl[ \int_{0}^{\alpha(t)}\bigl(\alpha^{\beta}(t)-s^{\beta } \bigr)^{p_{i}(\gamma-1)} s^{p_{i}(\xi-1)}\,ds \biggr]^{1/p_{i}} \biggl[ \int_{0}^{\alpha (t)}f_{1}^{q_{i}}(s)u^{q_{i}}(s) \,ds \biggr]^{1/q_{i}} \\ &{}+ \biggl[ \int_{0}^{\alpha(t)}\bigl(\alpha^{\beta}(t)-s^{\beta} \bigr)^{p_{i}(\gamma -1)} s^{p_{i}(\xi-1)}\,ds \biggr]^{1/p_{i}} \\ &{}\times\biggl[ \int_{0}^{\alpha(t)} \biggl(f_{2}(s) \int^{s}_{0} f_{3}(\tau)u(\tau)\,d\tau \biggr)^{q_{i}} \,ds \biggr]^{1/q_{i}}. \end{aligned}$$

Then $z(t)$ is a nondecreasing function, and $u(t)\le z(t)$, from (17), we have

$$\begin{aligned} z(t) \le& a(t)+ \biggl[ \int_{0}^{\alpha(t)}\bigl(\alpha^{\beta}(t)-s^{\beta } \bigr)^{p_{i}(\gamma-1)} s^{p_{i}(\xi-1)}\,ds \biggr]^{1/p_{i}} \biggl[ \int_{0}^{\alpha (t)}f_{1}^{q_{i}}(s)z^{q_{i}}(s) \,ds \biggr]^{1/q_{i}} \\ &{}+ \biggl[ \int_{0}^{\alpha(t)}\bigl(\alpha^{\beta}(t)-s^{\beta} \bigr)^{p_{i}(\gamma -1)} s^{p_{i}(\xi-1)}\,ds \biggr]^{1/p_{i}} \biggl[ \int_{0}^{\alpha(t)} \biggl(f_{2}(s) \int^{s}_{0} f_{3}(\tau)z(\tau)\,d\tau \biggr)^{q_{i}} \,ds \biggr]^{1/q_{i}} \\ \le& a(t)+ \biggl[ \int_{0}^{\alpha(t)}\bigl(\alpha^{\beta}(t)-s^{\beta } \bigr)^{p_{i}(\gamma-1)} s^{p_{i}(\xi-1)}\,ds \biggr]^{1/p_{i}} \biggl[ \int_{0}^{\alpha (t)}f_{1}^{q_{i}}(s)z^{q_{i}}(s) \,ds \biggr]^{1/q_{i}} \\ &{}+ \biggl[ \int_{0}^{\alpha(t)}\bigl(\alpha^{\beta}(t)-s^{\beta} \bigr)^{p_{i}(\gamma -1)} s^{p_{i}(\xi-1)}\,ds \biggr]^{1/p_{i}} \\ &{}\times \biggl[ \int_{0}^{\alpha(t)} \biggl(f_{2}(s) \int^{s}_{0} f_{3}(\tau)\,d\tau \biggr)^{q_{i}} z^{q_{i}}(s)\,ds \biggr]^{1/q_{i}}. \end{aligned}$$

Using the discrete Jensen inequality (6) in Lemma 2 with $n = 3$, $m = q_{i}$, we obtain

18 $$\begin{aligned} z^{q_{i}}(t) \le&3^{q_{i}-1}a^{q_{i}}(t)+3^{q_{i}-1} \biggl[ \int_{0}^{\alpha (t)}\bigl(\alpha^{\beta}(t)-s^{\beta} \bigr)^{p_{i}(\gamma-1)} s^{p_{i}(\xi-1)}\,ds \biggr]^{q_{i}/p_{i}} \int_{0}^{\alpha(t)}f_{1}^{q_{i}}(s)z^{q_{i}}(s) \,ds \\ &{}+3^{q_{i}-1} \biggl[ \int_{0}^{\alpha(t)}\bigl(\alpha^{\beta}(t)-s^{\beta } \bigr)^{p_{i}(\gamma-1)} s^{p_{i}(\xi-1)}\,ds \biggr]^{q_{i}/p_{i}} \\ &{}\times \int_{0}^{\alpha(t)} \biggl(f_{2}(s) \int^{s}_{0}f_{3}(\tau)\,d\tau \biggr)^{q_{i}}z^{q_{i}}(s) \,ds. \end{aligned}$$

Using Lemma 3, the inequality (18) can be restated as

19 $$\begin{aligned} z^{q_{i}}(t) \le&3^{q_{i}-1}a^{q_{i}}(t)+3^{q_{i}-1} \bigl(M_{i}\alpha^{\theta _{i}}(t) \bigr)^{q_{i}/p_{i}} \\ &{}\times \int_{0}^{\alpha(t)} \biggl[f_{1}^{q_{i}}(s) + \biggl(f_{2}(s) \int^{s}_{0} f_{3}(\tau)\,d\tau \biggr)^{q_{i}} \biggr]z^{q_{i}}(s) \,ds, \end{aligned}$$

for $t\in{\mathbf{R}_{+}}$, where

$$\begin{aligned}& M_{i} = \frac{1}{\beta}B \biggl[\frac{p_{i}(\xi-1)+1}{\beta},p_{i}( \gamma -1)+1 \biggr], \\& \theta_{i} = p_{i}\bigl[\beta(\gamma-1)+\xi-1 \bigr]+1\ge0, \end{aligned}$$

for $i = 1, 2$. Applying Lemma 4 to (19), we obtain

20 $$ u^{q_{i}}(t)\le z^{q_{i}}(t) \le \tilde{a}_{i}(t)+\frac{\tilde{b}_{i}(t)}{\tilde{e}_{i}(\alpha(t))} \int _{0}^{\alpha(t)}\tilde{h}_{i}(s) \tilde{a}_{i}(s)\tilde{e}_{i}(s)\,ds,\quad i=1,2, t\in{ \mathbf{R}_{+}}, $$

where

$$\begin{aligned}& \tilde{a}_{i}(t) = 3^{q_{i}-1}a^{q_{i}}(t), \\& \tilde{b}_{i}(t) = 3^{q_{i}-1} \bigl(M_{i} \alpha^{\theta_{i}}(t) \bigr)^{q_{i}/p_{i}}, \\& \tilde{h}_{i}(t) = f_{1}^{q_{i}}(s)+ \biggl(f_{2}(s) \int^{s}_{0} f_{3}(\tau)\,d\tau \biggr)^{q_{i}}, \\& \tilde{e}_{i}(t) = \exp \biggl(- \int_{0}^{t}\tilde{h}_{i}(s)\tilde {b}_{i}(s)\,ds \biggr), \end{aligned}$$

for $i=1,2$. Substituting $p_{1}=1/\gamma,q_{1}=1/(1-\gamma)$ and $p_{2}=(1+4\gamma )/(1+3\gamma)$, $q_{2}=(1+4\gamma)/\gamma$ to (20), respectively, we can get the desired estimations (15) and (16). This completes the proof. □

### Theorem 2

*Let* $u(t) $ *is a nonnegative piecewise continuous function with discontinuous of the first kind in the points* $t_{i}$ ($t_{0}< t_{1}< t_{2}<\cdots$, $\lim_{i\rightarrow\infty} t_{i}=\infty$), $a(t), f(t)\in C(\mathbf{R}_{+},\mathbf{R}_{+})$, $a(t)\ge1 $, *and let* $\alpha(t)$ *be a continuous*, *differentiable and increasing function on* $[t_{0},+\infty)$ *with* $\alpha(t)\le t$, $\alpha(t_{i})= t_{i}$, $i=0,1,2,\ldots $ . *Let* *p*, *β*, *γ* *be positive constants*, $\beta_{i}\in[0,\infty)$. *If* $u(t)$ *satisfies the inequality* (2), *then we have*

21 $$ u(t) \le \biggl(\tilde{a}_{i}(t)+\frac{1}{\tilde{e}_{i}(\alpha(t))} \int _{t_{i}}^{\alpha(t)}\tilde{h}(s)\tilde{a}_{i}(s) \tilde{e}_{i}(s)\,ds \biggr)^{1-\gamma},\quad t \in[t_{i},t_{i+1}),i=0,1,2,\ldots, $$

*where*

$$\begin{aligned}& \tilde{a}_{i}(t) = A_{i}^{\frac{1}{1-\gamma}}(t), \quad t\in [t_{i},t_{i+1}),i=0,1,2,\ldots, \\& A_{i}(t) = a(t)+\sum_{j=1}^{i} \int_{t_{j-1}}^{\alpha(t_{j})}\bigl(t^{\beta }-s^{\beta} \bigr)^{\gamma-1}f(s)u(s) \biggl[u^{2}(s)+ \int^{s}_{t_{0}}g(\tau)u(\tau )\,d\tau \biggr]^{p}\,ds \\& \hphantom{A_{i}(t) ={}}{}+\sum_{j=1}^{i} \beta_{j}u(t_{j}-0),\quad i=0,1,2,\ldots, \\& \tilde{h}(t) = \bigl(t^{\beta}-s^{\beta} \bigr)^{\gamma-1}f(t)\Omega\biggl(\frac{\alpha ^{-1}(s)}{\alpha'(s)}\biggr), \\& \tilde{e}_{i}(t) = \exp \biggl(- \int_{t_{i}}^{t}\tilde{h}(s)\,ds \biggr). \end{aligned}$$

### Proof

Firstly, we consider the case $t\in[t_{0},t_{1})$, denoting

22 $$ v(t)=a(t)+ \int_{t_{0}}^{\alpha(t)}\bigl(t^{\beta}-s^{\beta} \bigr)^{\gamma -1}f(s)u(s) \biggl[u^{2}(s)+ \int^{s}_{t_{0}}g(\tau)u(\tau)\,d\tau \biggr]^{p}\,ds, $$

then $v(t)$ is a nonnegative and nondecreasing continuous function, and

23 $$ u(t)\le v(t),\qquad v(t_{0})=a(t_{0}). $$

Differentiating (22) with respect to *t*, we have

24 $$\begin{aligned} v'(t) =&a'(t)+\alpha'(t) \bigl(t^{\beta}-\alpha^{\beta}(t)\bigr)^{\gamma-1}f\bigl(\alpha (t)\bigr)u\bigl(\alpha(t)\bigr) \biggl[u^{2}\bigl(\alpha(t)\bigr)+ \int^{\alpha(t)}_{t_{0}}g(s)u(s)\,ds \biggr]^{p} \\ \le& a'(t)+\alpha'(t) \bigl(t^{\beta}- \alpha^{\beta}(t)\bigr)^{\gamma-1}f\bigl(\alpha (t)\bigr)v\bigl( \alpha(t)\bigr) \biggl[v^{2}\bigl(\alpha(t)\bigr)+ \int^{\alpha(t)}_{t_{0}}g(s)v(s)\,ds \biggr]^{p}. \end{aligned}$$

Let

25 $$ \Gamma(t)=v^{2}\bigl(\alpha(t)\bigr)+ \int^{\alpha(t)}_{t_{0}} g(s)v(s)\,ds, $$

then $\Gamma(t)$ is a nonnegative and nondecreasing function, and $\Gamma(t_{0})= a^{2}(t_{0})$, since $a(t)\ge1$, we can conclude that $v(t)\le\Gamma(t)$, differentiating (25), from (24), we obtain

26 $$\begin{aligned} \Gamma'(t) =& 2v\bigl(\alpha(t)\bigr)v'\bigl( \alpha(t)\bigr)\alpha'(t)+\alpha'(t)g\bigl(\alpha (t) \bigr)v\bigl(\alpha(t)\bigr) \\ \le& 2\Gamma\bigl(\alpha(t)\bigr)\alpha'(t) \bigl(a'(t)+ \alpha'(t) \bigl(t^{\beta}-\alpha ^{\beta}(t) \bigr)^{\gamma-1}f\bigl(\alpha(t)\bigr)\Gamma\bigl(\alpha(t)\bigr) \Gamma^{p}(t) \bigr) \\ &{}+\alpha'(t)g\bigl(\alpha(t)\bigr)\Gamma\bigl(\alpha(t)\bigr) \\ \le& 2\Gamma(t)\alpha'(t) \bigl(a'(t)+ \alpha'(t) \bigl(t^{\beta}-\alpha^{\beta }(t) \bigr)^{\gamma-1}f\bigl(\alpha(t)\bigr)\Gamma(t)\Gamma^{p}(t) \bigr) \\ &{}+\alpha'(t)g\bigl(\alpha(t)\bigr)\Gamma(t). \end{aligned}$$

From (26), we have

27 $$\begin{aligned} \Gamma^{-(p+2)}\Gamma'(t) \le& \Gamma^{-(p+1)}(t) \bigl(2\alpha'(t)a'(t)+ \alpha'(t)g\bigl(\alpha(t)\bigr) \bigr) \\ &{}+2\bigl(\alpha'(t) \bigr)^{2}\bigl(t^{\beta}-\alpha^{\beta}(t) \bigr)^{\gamma-1}f\bigl(\alpha(t)\bigr). \end{aligned}$$

Let $\eta(t)=\Gamma^{-(p+1)}(t)$, then $\eta'(t)=-(p+1)\Gamma ^{-(p+2)}\Gamma'(t)$, (27) can be restated as

28 $$\begin{aligned}& \eta'(t)+(p+1)\eta(t) \bigl(2\alpha'(t)a'(t)+ \alpha'(t)g\bigl(\alpha(t)\bigr) \bigr) \\& \quad \ge-2(p+1) \bigl(\alpha'(t)\bigr)^{2} \bigl(t^{\beta}-\alpha^{\beta}(t)\bigr)^{\gamma -1}f\bigl( \alpha(t)\bigr). \end{aligned}$$

Multiplying by $\exp ((p+1)\int_{t_{0}}^{\alpha(t)}(2a'(\alpha ^{-1}(s))+g(s))\,ds )$ on both sides of (28), we have

29 $$ \begin{aligned}[b] &\biggl[\eta(t)\exp \biggl((p+1) \int_{t_{0}}^{\alpha(t)}\bigl(2a'\bigl(\alpha ^{-1}(s)\bigr)+g(s)\bigr)\,ds \biggr) \biggr]' \\ &\quad \ge-2(p+1) \bigl(\alpha'(t)\bigr)^{2} \bigl(t^{\beta}-\alpha^{\beta}(t)\bigr)^{\gamma -1}f\bigl( \alpha(t)\bigr) \\ &\qquad {}\times\exp \biggl((p+1) \int_{t_{0}}^{\alpha(t)}\bigl(2a'\bigl( \alpha^{-1}(s)\bigr)+g(s)\bigr)\,ds \biggr), \end{aligned} $$

integrating both sides of (29) from $t_{0}$ to *t*, we obtain

30 $$\begin{aligned}& \eta(t)\exp \biggl((p+1) \int_{t_{0}}^{\alpha(t)}\bigl(2a'\bigl(\alpha ^{-1}(s)\bigr)+g(s)\bigr)\,ds \biggr)-\eta(t_{0}) \\& \quad \ge-2(p+1) \bigl(\alpha'(t)\bigr)^{2} \bigl(t^{\beta}-\alpha^{\beta}(t)\bigr)^{\gamma -1}f\bigl( \alpha(t)\bigr) \\& \qquad {}\times\exp \biggl((p+1) \int_{t_{0}}^{\alpha(t)}\bigl(2a'\bigl( \alpha^{-1}(s)\bigr)+g(s)\bigr)\,ds \biggr) \\& \quad \ge \int^{\alpha(t)}_{t_{0}}-2(p+1) \bigl(t^{\beta}-s^{\beta}(t) \bigr)^{\gamma-1}f(s) \\& \qquad {}\times\exp \biggl((p+1) \int_{t_{0}}^{\alpha(s)}\bigl(2a'\bigl( \alpha^{-1}(\tau)\bigr)+g(\tau )\bigr)\,d\tau \biggr)\,ds, \end{aligned}$$

since $\eta(t_{0})=\Gamma^{-(p+1)}(t_{0})=a^{-2(p+1)}(t_{0})$, denoting $\Delta(t)=\exp ((p+1)\int_{t_{0}}^{\alpha(s)}(2a'(\alpha ^{-1}(\tau))+ g(\tau))\,d\tau )$, from (30), we have

31 $$ \eta(t)\ge \frac{1-2a^{2(p+1)(t_{0})}(p+1)\int^{\alpha(t)}_{t_{0}}(t^{\beta}-s^{\beta })^{\gamma-1}f(s) \Delta(s)}{ a^{2(p+1)}(t_{0})\Delta(t)}, $$

by $\eta(t)=\Gamma^{-(p+1)}(t)$, from (31), we have

32 $$ \Gamma^{p}(t) \le \biggl[\frac{a^{2(p+1)}(t_{0})\Delta(t)}{ 1-2a^{2(p+1)}(t_{0})(p+1)\int^{\alpha(t)}_{t_{0}}(t^{\beta}-s^{\beta })^{\gamma-1}f(s) \Delta(s)\,ds} \biggr]^{\frac{p}{p+1}}, $$

where $1-2a^{2(p+1)}(t_{0})(p+1)\int^{\alpha(t)}_{t_{0}}(t^{\beta}-s^{\beta })^{\gamma-1}f(s)\,ds>0$, setting

33 $$ \Omega(t) = \biggl[\frac{a^{2(p+1)}(t_{0})\Delta(t)}{ 1-2a^{2(p+1)}(t_{0})(p+1)\int^{\alpha(t)}_{t_{0}}(t^{\beta}-s^{\beta })^{\gamma-1}f(s)\Delta(s)\,ds} \biggr]^{\frac{p}{p+1}}, $$

from (24), (25), (32) and (33), we have

34 $$ v'(t) \le a'(t)+\alpha'(t) \bigl(t^{\beta}-\alpha^{\beta}(t)\bigr)^{\gamma-1}f\bigl(\alpha (t)\bigr)v\bigl(\alpha(t)\bigr)\Omega(t). $$

Integrating both side of (34) from $t_{0}$ to *t*, we get

35 $$\begin{aligned} \begin{aligned}[b] v(t) &\le a(t)+ \int^{t}_{t_{0}}\alpha'(s) \bigl(t^{\beta}-\alpha^{\beta}(s)\bigr)^{\gamma -1}f\bigl( \alpha(s)\bigr)v\bigl(\alpha(s)\bigr)\Omega(s)\,ds \\ &= a(t)+ \int^{\alpha(t)}_{t_{0}}\bigl(t^{\beta}-s^{\beta} \bigr)^{\gamma -1}f(s)v(s)\Omega \biggl(\frac{\alpha^{-1}(s)}{\alpha'(s)} \biggr) \,ds. \end{aligned} \end{aligned}$$

Equation (35) has the same form as Lemma 4, and the functions of (35) satisfy the conditions of Theorem 1. Consequently, by using a similar procedure to Lemma 4 and Theorem 1, we can get the desired estimations (21) for $t\in[t_{0},t_{1})$.

Next, let us consider the interval $[t_{1},t_{2})$, when $t\in[t_{1},t_{2})$, (2) can be restated as

36 $$\begin{aligned} u(t) \le& a(t)+ \int_{t_{0}}^{\alpha(t_{1})}\bigl(t^{\beta}-s^{\beta} \bigr)^{\gamma -1}f(s)u(s) \biggl[u^{2}(s)+ \int^{s}_{t_{0}}g(\tau)u(\tau)\,d\tau \biggr]^{p}\,ds \\ &{}+ \int_{t_{1}}^{\alpha(t)}\bigl(t^{\beta}-s^{\beta} \bigr)^{\gamma-1}f(s)u(s) \biggl[u^{2}(s)+ \int^{s}_{t_{1}}g(\tau)u(\tau)\,d\tau \biggr]^{p}\,ds+\beta_{1}u(t_{1}-0), \end{aligned}$$

setting

37 $$\begin{aligned}& A_{1}(t) = a(t)+ \int_{t_{0}}^{\alpha(t_{1})}\bigl(t^{\beta}-s^{\beta} \bigr)^{\gamma -1}f(s)u(s) \biggl[u^{2}(s)+ \int^{s}_{t_{0}}g(\tau)u(\tau)\,d\tau \biggr]^{p}\,ds+\beta _{1}u(t_{1}-0), \\& \Psi(t) = a(t)+ \int_{t_{0}}^{\alpha(t_{1})}\bigl(t^{\beta}-s^{\beta} \bigr)^{\gamma -1}f(s)u(s) \biggl[u^{2}(s)+ \int^{s}_{t_{0}}g(\tau)u(\tau)\,d\tau \biggr]^{p}\,ds \\& \hphantom{\Psi(t) ={}}{}+ \int_{t_{1}}^{\alpha(t)}\bigl(t^{\beta}-s^{\beta} \bigr)^{\gamma-1}f(s)u(s) \biggl[u^{2}(s)+ \int^{s}_{t_{0}}g(\tau)u(\tau)\,d\tau \biggr]^{p}\,ds+\beta_{1}u(t_{1}-0), \end{aligned}$$

then $\Psi(t)$ is a nonnegative and nondecreasing function, and

$$u(t)\le\Psi(t),\qquad u(t_{1})\le\Psi(t_{1})=A_{1}(t_{1}). $$

Differentiating with respect to *t* both sides of (37), we obtain

38 $$\begin{aligned} \Psi'(t) =& A_{1}'(t) + \alpha'(t) \bigl(t^{\beta}-\alpha(t)^{\beta} \bigr)^{\gamma-1}f\bigl(\alpha(t)\bigr)u\bigl(\alpha (t)\bigr) \biggl[u^{2}\bigl(\alpha(t)\bigr)+ \int^{\alpha(t)}_{t_{0}}g(s)u(s)\,ds \biggr]^{p} \\ \le& A_{1}'(t) +\alpha'(t) \bigl(t^{\beta}-\alpha(t)^{\beta}\bigr)^{\gamma-1}f\bigl( \alpha(t)\bigr)\Psi \bigl(\alpha(t)\bigr) \\ &{}\times \biggl[\Psi^{2}\bigl(\alpha(t) \bigr)+ \int^{\alpha(t)}_{t_{0}}g(s)\Psi(s)\,ds \biggr]^{p}, \end{aligned}$$

(38) has the same form of (24), and using a similar procedure for $t\in[t_{1},t_{2})$, we can get the desired estimations (21) for $t\in[t_{1},t_{2})$.

Consequently, by using a similar procedure for $t\in[t_{i},t_{i+1})$, we can get the desired estimations (21) for $t\in[t_{i},t_{i+1})$. Thus we complete the proof of Theorem 2. □

### Theorem 3

*Let* $u(t) $ *is a nonnegative piecewise continuous function with discontinuous of the first kind in the points* $t_{i}$ ($t_{0}< t_{1}< t_{2}<\cdots$, $\lim_{i\rightarrow\infty} t_{i}=\infty$), $a(t), f(t)\in C(\mathbf{R}_{+},\mathbf{R}_{+})$, $a(t)\ge1 $, *and let* $\alpha(t)$ *be a continuous*, *differentiable and increasing function on* $[t_{0},+\infty)$ *with* $\alpha(t)\le t$, $\alpha(t_{i})= t_{i}$, $i=0,1,2,\ldots $ . *Let* *p*, *q*, *m*, *n*, *ξ*, *β*, *γ* *be positive constants with* $p\ge m$, $p\ge n$, $q \in[0,1]$, $\beta_{i}\in[0,\infty)$. *If* $u(t)$ *satisfies the inequality* (3).

(1) *If* $\beta\in(0,1]$, $\gamma\in(1/2,1)$ *and* $\xi\ge3/2-\gamma$, *we have*

39 $$\begin{aligned}& u(t) \le \biggl[E_{i}(t)+ \biggl(\tilde{a}_{i}(t)+ \frac{\tilde{b}_{1}(t)}{ \tilde{e}_{i}(\alpha(t))} \int_{t_{i}}^{\alpha(t)}\tilde{h}_{i}(s)\tilde {a}_{i}(s)\tilde{e}_{i}(s)\,ds \biggr)^{1-\gamma} \biggr]^{1/p}, \\& \quad t\in[t_{i},t_{i+1}),i=0,1,2,\ldots, \end{aligned}$$

*where* $M_{1}$, $\theta_{1}$ *are the same as in Theorem* 1, *and*

$$\begin{aligned}& E_{0}(t) = a(t),\quad t\in[t_{0},t_{1}), \\& E_{i}(t) = a(t)+b(t)\sum_{j=0}^{i} \int_{t_{j}}^{\alpha(t_{i})}\bigl(\alpha^{\beta }(t)-s^{\beta} \bigr)^{\gamma-1} s^{\xi-1}f(s) \biggl[u^{m}(s)+ \int^{s}_{t_{j}}g(\tau )u^{n}(\tau)\,d\tau \biggr]^{q}\,ds \\& \hphantom{E_{i}(t) ={}}{}+\sum_{j=1}^{i} \beta_{j}u^{p}(t_{j}-0),\quad t \in[t_{i},t_{i+1}),i=1,2,\ldots, \\& \tilde{a}_{i}(t) = 3^{\frac{\gamma}{1-\gamma}}A_{i}^{\frac{1}{1-\gamma }}(t), \quad i=0,1,2,\ldots, \\& A_{i}(t) = b(t) \int_{t_{i}}^{\alpha(t)}\bigl(t^{\beta}-s^{\beta} \bigr)^{\gamma-1} s^{\xi-1}B_{i}(s)\,ds,\quad i=0,1,2, \ldots, \\& B_{i}(t) = f(t) \biggl[(1-q) +q \biggl(\frac{m}{p}E_{i}(t)+ \frac{p-m}{p} \biggr) \biggr] \\& \hphantom{B_{i}(t) ={}}{}+q f(t) \int^{t}_{t_{i}}g(\tau) \biggl[\frac{n}{p}E_{i}( \tau)+\frac{p-n}{p} \biggr]\,d\tau,\quad i=0,1,2,\ldots, \\& \tilde{b}_{1}(t) = \bigl(3M_{1}\alpha^{\theta_{1}}(t) \bigr)^{\frac{\gamma}{1-\gamma }}b^{\frac{1}{1-\gamma}}(t), \\& \tilde{e}_{i}(t) = \exp \biggl(- \int_{t_{i}}^{t}\tilde{h}_{i}(s)\tilde {b}_{1}(s)\,ds \biggr),\quad i=0,1,2,\ldots, \\& \tilde{h}_{i}(t) = g_{1}^{\frac{1}{1-\gamma}}(t)+ \biggl(g_{2}(t) \int ^{t}_{t_{i}}g_{3}(\tau)\,d\tau \biggr)^{\frac{1}{1-\gamma}}, \\& g_{1}(t) = \frac{mq}{p} f(t),\qquad g_{2}(t)=q f(t), \qquad g_{3}(t)=\frac{n}{p}g(t). \end{aligned}$$

(2) *If* $\beta\in(0,1]$, $\gamma\in(0,1/2]$ *and* $\xi> (1-2\gamma ^{2})/(1-\gamma^{2})$, *we have*

40 $$\begin{aligned}& u(t)\le \biggl[ E_{i}(t)+ \biggl(\tilde{a}_{i}(t)+ \frac{\tilde{b}_{2}(t)}{\tilde {e}_{i}(\alpha(t))} \int_{0}^{\alpha(t)} \tilde{h}_{i}(s) \tilde{a}_{i}(s)\tilde{e}_{i}(s)\,ds \biggr)^{\frac{\gamma }{1+4\gamma}} \biggr]^{1/p}, \\& \quad t\in[t_{i},t_{i+1}),i=0,1,2,\ldots, \end{aligned}$$

*where* $M_{2}$, $\theta_{2}$ *are the same as in Theorem* 1 *and* $E_{i}$, $A_{i}$, $B_{i}$, $h_{i}$, $i=0,1,2,\ldots$ , *are the same in* (1) *of Theorem* 3,

$$\begin{aligned}& \tilde{a}_{i}(t) = 3^{\frac{1+3\gamma}{\gamma}}A_{i}^{\frac{1+4\gamma }{\gamma}}(t), \quad i=0,1,2,\ldots, \\& \tilde{b}_{2}(t) = \bigl(3M_{2}\alpha^{\theta_{2}}(t) \bigr)^{\frac{1+3\gamma}{\gamma }}b^{\frac{1+4\gamma}{\gamma}}(t), \\& \tilde{e}_{i}(t) = \exp \biggl(- \int_{t_{i}}^{t}\tilde{h}_{i}(s)\tilde {b}_{2}(s)\,ds \biggr),\quad i=0,1,2,\ldots. \end{aligned}$$

### Proof

When $t\in[t_{0},t_{1})$, (3) can be restated as

41 $$ u^{p}(t) \le a(t)+b(t) \int_{t_{0}}^{\alpha(t)}\bigl(\alpha^{\beta}(t)-s^{\beta } \bigr)^{\gamma-1} s^{\xi-1}f(s) \biggl[u^{m}(s)+ \int^{s}_{t_{0}}g(\tau)u^{n}(\tau )\,d\tau \biggr]^{q}\,ds, $$

by Lemma 5, we obtain

42 $$ \biggl[u^{m}(s)+ \int^{s}_{t_{0}}g(\tau)u^{n}(\tau)\,d\tau \biggr]^{q}\le q \biggl[u^{m}(s)+ \int^{s}_{t_{0}}g(\tau)u^{n}(\tau)\,d\tau \biggr]+(1-q). $$

Substituting (42) into (41), we have

43 $$\begin{aligned} u^{p}(t) \le& a(t)+b(t) \int_{t_{0}}^{\alpha(t)}\bigl(\alpha^{\beta}(t)-s^{\beta } \bigr)^{\gamma-1} s^{\xi-1}f(s) \\ &{}\times \biggl[q \biggl(u^{m}(s) + \int^{s}_{t_{0}}g(\tau)u^{n}(\tau)\,d\tau \biggr)+(1-q) \biggr]\,ds. \end{aligned}$$

Define a function $w(t)$ by

44 $$\begin{aligned} w(t) =&b(t) \int_{t_{0}}^{\alpha(t)}\bigl(\alpha^{\beta}(t)-s^{\beta} \bigr)^{\gamma -1} s^{\xi-1}(1-q) f(s)\,ds \\ &{}+b(t) \int_{t_{0}}^{\alpha(t)}\bigl(\alpha^{\beta}(t)-s^{\beta} \bigr)^{\gamma-1} s^{\xi-1}q f(s)u^{m}(s)\,ds \\ &{}+b(t) \int_{t_{0}}^{\alpha(t)}\bigl(\alpha^{\beta}(t)-s^{\beta} \bigr)^{\gamma-1} s^{\xi-1}q f(s) \int^{s}_{t_{0}}g(\tau)u^{n}(\tau)\,d\tau \,ds, \end{aligned}$$

from (43) and (44), we have

45 $$ u^{p}(t) \le a(t)+w(t) \quad \mbox{or}\quad u(t)\le \bigl(a(t)+w(t) \bigr)^{1/p}. $$

By Lemma 5 and (45), we obtain

46 $$\begin{aligned}& u^{m}(t) \le \bigl(a(t)+w(t) \bigr)^{m/p}\le \frac{m}{p}\bigl(a(t)+w(t)\bigr)+\frac {p-m}{p}, \end{aligned}$$

47 $$\begin{aligned}& u^{n}(t) \le \bigl(a(t)+w(t) \bigr)^{n/p}\le \frac{n}{p}\bigl(a(t)+w(t)\bigr)+\frac{p-n}{p}. \end{aligned}$$

Substituting the inequality (46) and (47) into (44) we have

48 $$\begin{aligned} w(t) \le&b(t) \int_{t_{0}}^{\alpha(t)}\bigl(\alpha^{\beta}(t)-s^{\beta} \bigr)^{\gamma -1} s^{\xi-1}(1-q) f(s)\,ds \\ &{}+b(t) \int_{t_{0}}^{\alpha(t)}\bigl(\alpha^{\beta}(t)-s^{\beta} \bigr)^{\gamma-1} s^{\xi-1}q f(s) \biggl[\frac{m}{p} \bigl(a(s)+w(s)\bigr)+\frac{p-m}{p} \biggr]\,ds \\ &{}+b(t) \int_{t_{0}}^{\alpha(t)}\bigl(\alpha^{\beta}(t)-s^{\beta} \bigr)^{\gamma-1} s^{\xi-1}q f(s) \int^{s}_{t_{0}}g(\tau) \biggl[\frac{n}{p} \bigl(a(\tau)+w(\tau)\bigr)+\frac{p-n}{p} \biggr]\,d\tau \,ds \\ \le&b(t) \int_{t_{0}}^{\alpha(t)}\bigl(\alpha^{\beta}(t)-s^{\beta} \bigr)^{\gamma-1} s^{\xi-1}f(s) \biggl[(1-q) +q \biggl( \frac{m}{p}a(s)+\frac{p-m}{p} \biggr) \biggr]\,ds \\ &{}+b(t) \int_{t_{0}}^{\alpha(t)}\bigl(\alpha^{\beta}(t)-s^{\beta} \bigr)^{\gamma-1} s^{\xi-1}q f(s) \int^{s}_{t_{0}}g(\tau) \biggl[\frac{n}{p}a( \tau)+\frac {p-n}{p} \biggr]\,d\tau \,ds \\ &{}+b(t) \int_{t_{0}}^{\alpha(t)}\bigl(\alpha^{\beta}(t)-s^{\beta} \bigr)^{\gamma-1} s^{\xi-1}\frac{mq}{p}f(s)w(s)\,ds \\ &{}+b(t) \int_{t_{0}}^{\alpha(t)}\bigl(\alpha^{\beta}(t)-s^{\beta} \bigr)^{\gamma-1} s^{\xi-1}q f(s) \int^{s}_{t_{0}}\frac{n}{p}g(\tau)w(\tau)\,d\tau \,ds \\ \le&b(t) \int_{t_{0}}^{\alpha(t)}\bigl(\alpha^{\beta}(t)-s^{\beta} \bigr)^{\gamma-1} s^{\xi-1}B_{0}(s)\,ds+b(t) \int_{t_{0}}^{\alpha(t)}\bigl(t^{\beta}-s^{\beta } \bigr)^{\gamma-1} s^{\xi-1}g_{1}(s)w(s)\,ds \\ &{}+b(t) \int_{t_{0}}^{\alpha(t)}\bigl(\alpha^{\beta}(t)-s^{\beta} \bigr)^{\gamma-1} s^{\xi-1}g_{2}(s) \int^{s}_{t_{0}}g_{3}(\tau)w(\tau)\,d\tau \,ds, \\ =&A_{0}(t)+b(t) \int_{t_{0}}^{\alpha(t)}\bigl(\alpha^{\beta}(t)-s^{\beta } \bigr)^{\gamma-1} s^{\xi-1}g_{1}(s)w(s)\,ds \\ &{}+b(t) \int_{t_{0}}^{\alpha(t)}\bigl(\alpha^{\beta}(t)-s^{\beta} \bigr)^{\gamma-1} s^{\xi-1}g_{2}(s) \int^{s}_{t_{0}}g_{3}(\tau)w(\tau)\,d\tau \,ds, \end{aligned}$$

where

$$\begin{aligned}& A_{0}(t) = b(t) \int_{t_{0}}^{\alpha(t)}\bigl(t^{\beta}-s^{\beta} \bigr)^{\gamma-1} s^{\xi-1}B_{0}(s)\,ds, \\& B_{0}(t) = f(t) \biggl[(1-q) +q \biggl(\frac{m}{p}a(t)+ \frac{p-m}{p} \biggr) \biggr] \\& \hphantom{B_{0}(t) = {}}{}+q f(t) \int^{t}_{t_{0}}g(\tau) \biggl[\frac{n}{p}a( \tau)+\frac{p-n}{p} \biggr]\,d\tau, \\& g_{1}(t) = \frac{mq}{p} f(t),\qquad g_{2}(t)=q f(t), \qquad g_{3}(t)=\frac {n}{p}g(t). \end{aligned}$$

Since (48) have the same form as (1) and the functions of (48) satisfy the conditions of Theorem 1, applying Theorem 1 to (48), considering equation (45), we can get the desired estimations (39) and (40) for $t\in[t_{0},t_{1})$.

Then, when $t\in[t_{1},t_{2})$, (3) can be restated as

$$\begin{aligned} u^{p}(t) \le&a(t)+b(t) \int_{t_{0}}^{\alpha(t_{1})}\bigl(\alpha^{\beta}(t)-s^{\beta } \bigr)^{\gamma-1} s^{\xi-1}f(s) \biggl[u^{m}(s)+ \int^{s}_{t_{0}}g(\tau)u^{n}(\tau )\,d\tau \biggr]^{q}\,ds \\ &{}+\beta_{1}u^{p}(t_{1}-0) +b(t) \int_{t_{1}}^{\alpha(t)}\bigl(\alpha^{\beta}(t)-s^{\beta} \bigr)^{\gamma-1} s^{\xi-1}f(s) \\ &{}\times \biggl[u^{m}(s)+ \int^{s}_{t_{1}}g(\tau)u^{n}(\tau)\,d\tau \biggr]^{q}\,ds. \end{aligned}$$

Let

$$\begin{aligned} E_{1}(t) =&a(t)+b(t) \int_{t_{0}}^{\alpha(t_{1})}\bigl(\alpha^{\beta}(t)-s^{\beta } \bigr)^{\gamma-1} s^{\xi-1}f(s) \biggl[u^{m}(s)+ \int^{s}_{t_{0}}g(\tau)u^{n}(\tau )\,d\tau \biggr]^{q}\,ds \\ &{}+\beta_{1}u^{p}(t_{1}-0), \end{aligned}$$

then we have

49 $$ u^{p}(t) \le E(t) +b(t) \int_{t_{1}}^{\alpha(t)}\bigl(\alpha^{\beta}(t)-s^{\beta} \bigr)^{\gamma-1} s^{\xi-1}f(s) \biggl[u^{m}(s)+ \int^{s}_{t_{1}}g(\tau)u^{n}(\tau)\,d\tau \biggr]^{q}\,ds. $$

From (49), we can conclude that the estimates (39)and (40) are valid for $t\in[t_{1},t2)$. Consequently, by using a similar procedure for $t\in[t_{i},t_{i+1})$, we complete the proof of theorem. □

## Some applications

### Example 1

Consider the following Volterra type retarded weakly singular integral equations:

50 $$ y^{p}(t)- \int^{\alpha(t)}_{t_{0}}\bigl(\alpha^{\beta}(t)-s^{\beta}\bigr)^{\gamma -1}s^{\beta(1+\delta)-1} \biggl[y(s)+ \int^{s}_{t_{0}}g(\tau)y(\tau)\,d\tau \biggr]^{q}\,ds=h(t), $$

which arises very often in various problems, especial describing physical processes with aftereffects. Ma and Pečarić [18] discussed the case $\alpha(t)=t$, $g(t)\equiv0$ in (50).

### Theorem 4

*Let* $y(t)$, $g(t)$ *and* $h(t)$ *be continuous functions on* $[0,+\infty)$, *and let* $\alpha(t)$ *be continuous*, *differentiable and increasing functions on* $[0,+\infty)$ *with* $\alpha(t)\le t$, $\alpha(t_{0})= t_{0}$. *Let* *p*, *q*, *β*, *γ*, *δ* *be positive constants with* $p\ge q$. *Assume* $y(t)$ *satisfies equation* (50).

(1) *If* $\beta\in(0,1]$, $\gamma\in(1/2,1)$ *and* $\beta(1+\delta)\ge 3/2-\gamma$, *we have*

51 $$ \bigl\vert y(t) \bigr\vert \le \biggl[ \bigl\vert h(t) \bigr\vert + \biggl(\tilde{a}_{1}(t)+\frac{\tilde{b}_{1}(t)}{\tilde {e}_{1}(\alpha(t))} \int_{t_{0}}^{\alpha(t)} \tilde{h}_{1}(s) \tilde{a}_{1}(s)\tilde{e}_{1}(s)\,ds \biggr)^{1-\gamma} \biggr]^{1/p},\quad t\in{\mathbf{R}_{+}}, $$

*where*

$$\begin{aligned}& \tilde{a}_{1}(t) = 3^{\frac{\gamma}{1-\gamma}} \int_{t_{0}}^{\alpha (t)}A_{1}^{\frac{1}{1-\gamma}}(s)\,ds, \\& \tilde{b}_{1}(t) = \bigl(3M_{1}\alpha^{\theta_{1}}(t) \bigr)^{\frac{\gamma }{1-\gamma}}, \\& \tilde{h}_{1}(t) = A_{2}^{\frac{1}{1-\gamma}}(t)+ \biggl(A_{3}(t) \int ^{t}_{t_{0}}A_{4}(\tau)\,d\tau \biggr)^{\frac{1}{1-\gamma}}, \\& \tilde{e}_{1}(t) = \exp \biggl(- \int_{0}^{t}\tilde{h}_{1}(s)\tilde {b}_{1}(s)\,ds \biggr), \\& M_{1} = \frac{1}{\beta}B \biggl[\frac{\gamma+\xi-1}{\beta\gamma}, \frac {2\gamma-1}{\gamma} \biggr], \\& \theta_{1} = \frac{1}{\gamma}\bigl[\beta(\gamma-1)+\xi-1\bigr]+1, \\& A_{1}(t) = (1-q) +q \biggl(\frac{1}{p} \bigl\vert h(t) \bigr\vert +\frac{p-1}{p} \biggr) \\& \hphantom{A_{1}(t) = {}}{} +q K^{q-1} \int^{t}_{0} \bigl\vert g(\tau) \bigr\vert \biggl[\frac{1}{p} \bigl\vert h(\tau) \bigr\vert +\frac{p-1}{p} \biggr]\,d\tau, \\& A_{2}(t) = \frac{q}{p} , \qquad A_{3}(t)=q K^{q-1},\qquad A_{4}(t)=\frac{1}{p} \bigl\vert g(t) \bigr\vert . \end{aligned}$$

(2) *If* $\beta\in(0,1]$, $\gamma\in(0,1/2]$ *and* $\xi> (1-2\gamma ^{2})/(1-\gamma^{2})$, *we have*

52 $$ \bigl\vert y(t) \bigr\vert \le \biggl[ \bigl\vert h(t) \bigr\vert + \biggl(\tilde{a}_{2}(t)+\frac{\tilde{b}_{2}(t)}{\tilde {e}_{2}(\alpha(t))} \int_{t_{0}}^{\alpha(t)}\tilde{h}_{2}(s) \tilde{a}_{2}(s)\tilde{e}_{2}(s)\,ds \biggr)^{\frac{\gamma}{1+4\gamma}} \biggr]^{1/p},\quad t\in{\mathbf{R}_{+}}, $$

*where*

$$\begin{aligned}& \tilde{a}_{2}(t) = \bigl(3M_{2}\alpha^{\theta_{2}}(t) \bigr)^{\frac{1+3\gamma }{\gamma}} \int_{t_{0}}^{\alpha(t)}A_{1}^{\frac{1+4\gamma}{\gamma}}(s)\,ds, \\& \tilde{b}_{2}(t) = \bigl(3M_{2}\alpha^{\theta_{2}}(t) \bigr)^{\frac{1+3\gamma }{\gamma}}, \\& \tilde{h}_{2}(t) = A_{2}^{\frac{1+4\gamma}{\gamma}}(s)+ \biggl(A_{3}(s) \int ^{s}_{t_{0}}A_{4}(\tau)\,d\tau \biggr)^{\frac{1+4\gamma}{\gamma}}, \\& \tilde{e}_{2}(t) = \exp \biggl(- \int_{t_{0}}^{t}\tilde{h}_{2}(s)\tilde {b}_{2}(s)\,ds \biggr), \\& M_{2} = \frac{1}{\beta}B \biggl[\frac{\xi(1+4\gamma)-\gamma}{\beta(1+3\gamma )}, \frac{4\gamma^{2}}{1+3\gamma} \biggr], \\& \theta_{2} = \frac{1+4\gamma}{1+3\gamma}\bigl[\beta(\gamma-1)+\xi-1\bigr]+1. \end{aligned}$$

### Proof

From (50), we have

53 $$ \bigl\vert y(t) \bigr\vert ^{p}\le \bigl\vert h(t) \bigr\vert + \int^{\alpha(t)}_{t_{0}}\bigl(\alpha^{\beta}(t)-s^{\beta}\bigr)^{\gamma-1}s^{\beta(1+\delta)-1} \biggl[ \bigl\vert y(s) \bigr\vert + \int^{s}_{t_{0}} \bigl\vert g(\tau ) \bigr\vert \bigl\vert y(\tau) \bigr\vert \,d\tau \biggr]^{q}\,ds. $$

Applying Theorem 3 for $t\in[t_{0},t_{1})$ (with $m=n=1$, $a(t)=|h(t)|$, $b(t)=|\lambda|t^{-\beta\delta}/\Gamma(\gamma)$, $\xi =\beta(1+\delta)$) to (53), we obtain the desired estimations (51) and (52). □

### Example 2

Consider the following impulsive differential system:

54 $$\begin{aligned}& \frac{d(x(t))}{dt} = F(t,x),\quad t\neq t_{i}, t \in[t_{0},\infty), \end{aligned}$$

55 $$\begin{aligned}& \Delta(x) |_{t=t_{i}} = \beta_{i} x(t_{i}-0), \\& x(t_{0}) = x_{0}, \end{aligned}$$

where $0\leq t_{0}< t_{1}< t_{2}<\cdots$, $\lim_{i\rightarrow\infty}t_{i}=\infty$, $x_{0}>0$ is a constant, $F(t,x)$ is continuous with respect to *t* and *x* on $[t_{0},\infty) \times(0,+\infty)$. Suppose $F(s,x)$ satisfies

56 $$ F(s,x)\le\bigl(t^{\beta}-s^{\beta} \bigr)^{\gamma-1}f(s)\sqrt{x(s)}, $$

where $f(t)\in C(\mathbf{R}_{+},\mathbf{R}_{+})$, $\beta\in(0,1]$, $\gamma \in(1/2,1)$.

Then the impulsive differential system (54) and (55) are equivalent to the integral equation

57 $$ x(t)=x_{0}+ \int^{t}_{t_{0}}F\bigl(s,x(s)\bigr)\,ds+\sum _{t_{0}< t_{i}< t}\beta_{i} x(t_{i}-0). $$

By using the condition (56), from (57), we have

58 $$ \bigl\vert x(t) \bigr\vert \le x_{0}+ \int^{t}_{t_{0}}\bigl(t^{\beta}-s^{\beta} \bigr)^{\gamma-1}f(s)\sqrt {x(s)}\,ds+\sum_{t_{0}< t_{i}< t} \beta_{i} \bigl\vert x(t_{i}-0) \bigr\vert . $$

Let $u(t)=|x(t)|$, from (58), we get

59 $$ u(t) \le x_{0}+ \int^{t}_{t_{0}}\bigl(t^{\beta}-s^{\beta} \bigr)^{\gamma-1}f(s)\sqrt {u(s)}\,ds+\sum_{t_{0}< t_{i}< t} \beta_{i}u(t_{i}-0). $$

By Lemma 5, we have

60 $$ u^{\frac{1}{2}}(t)\le\frac{1}{2}u(t)+\frac{1}{2}. $$

Substituting (60) to (59), we have

61 $$\begin{aligned} u(t) \le& x_{0}+ \int^{t}_{t_{0}}\bigl(t^{\beta}-s^{\beta} \bigr)^{\gamma-1}f(s) \biggl(\frac {1}{2}u(s)+\frac{1}{2}\biggr) \,ds+\sum_{t_{0}< t_{i}< t}\beta_{i}u(t_{i}-0) \\ \le& x_{0}+ \int^{t}_{t_{0}}\bigl(t^{\beta}-s^{\beta} \bigr)^{\gamma-1}\frac{f(s)}{2}u(s)\,ds + \int^{t}_{t_{0}}\bigl(t^{\beta}-s^{\beta} \bigr)^{\gamma-1}\frac{f(s)}{2}\,ds +\sum_{t_{0}< t_{i}< t} \beta_{i}u(t_{i}-0) \\ \le& a(t)+ \int^{t}_{t_{0}}\bigl(t^{\beta}-s^{\beta} \bigr)^{\gamma-1}\frac {f(s)}{2}u(s)\,ds+\sum_{t_{0}< t_{i}< t} \beta_{i}u(t_{i}-0), \end{aligned}$$

where $a(t)=x_{0}+\int^{t}_{t_{0}}(t^{\beta}-s^{\beta})^{\gamma-1}\frac{f(s)}{2}\,ds$.

We see that (61) is the particular form of (3), and the functions of (54) satisfy the conditions of Theorem 3, using the result of Theorem 3, we can conclude that we have the estimated solutions for the impulsive system

$$\begin{aligned}& u(t) \le E_{i}(t)+ \biggl(\tilde{a}_{i}(t)+ \frac{\tilde{b}(t)}{ \tilde{e}_{i}(t)} \int_{t_{i}}^{t}\tilde{h}(s)\tilde{a}_{i}(s) \tilde {e}_{i}(s)\,ds \biggr)^{1-\gamma}, \\& \quad t\in[t_{i},t_{i+1}),i=0,1,2,\ldots, \end{aligned}$$

where $M_{1}$, $\theta_{1}$ are the same as in Theorem 3, and

$$\begin{aligned}& E_{0}(t) = a(t),\quad t\in[t_{0},t_{1}), \\& E_{i}(t) = a(t)+\sum_{j=0}^{i} \int_{t_{j}}^{t_{i}}\bigl(\alpha^{\beta}(t)-s^{\beta } \bigr)^{\gamma-1} f(s)\sqrt{u(s)}\,ds \\& \hphantom{E_{i}(t) ={}}{}+\sum_{j=1}^{i} \beta_{j}u(t_{j}-0),\quad t\in[t_{i},t_{i+1}),i=1,2, \ldots, \\& \tilde{a}_{i}(t) = 2^{\frac{\gamma}{1-\gamma}}A_{i}^{\frac{1}{1-\gamma }}(t), \quad i=0,1,2,\ldots, \\& A_{i}(t) = \int_{t_{i}}^{t}\bigl(t^{\beta}-s^{\beta} \bigr)^{\gamma-1} B_{i}(s)\,ds,\quad i=0,1,2,\ldots, \\& B_{i}(t) = f(t) \biggl(\frac{1}{2} +\frac{1}{2}E_{i}(t) \biggr),\quad i=0,1,2,\ldots, \\& \tilde{b}(t) = \bigl(2M_{1}\alpha^{\theta_{1}}(t) \bigr)^{\frac{\gamma}{1-\gamma}}, \\& \tilde{e}_{i}(t) = \exp \biggl(- \int_{t_{i}}^{t}\tilde{h}_{i}(s)\tilde {b}_{1}(s)\,ds \biggr),\quad i=0,1,2,\ldots, \\& \tilde{h}(t) = g_{1}^{\frac{1}{1-\gamma}}(t),\qquad g_{1}(t)= \frac{1}{2}f(t) . \end{aligned}$$

## Conclusion

In this paper, we generalized the weakly singular integral inequality. The first inequality was a generally weak singular type, the second inequality was a like-weakly singular type with discontinuous functions, the third inequality was a type of weakly singular integral inequality with impulsive. We used analytical methods, reducing the inequality with the known results in the lemma, and the estimations of the upper bound of the unknown functions were given. The results were applied to the weakly singular integral equation and the impulsive differential system.
